# (3-Amino­phen­yl)methanol

**DOI:** 10.1107/S1600536811029163

**Published:** 2011-07-23

**Authors:** Richard Betz, Thomas Gerber, Eric Hosten

**Affiliations:** a Nelson Mandela Metropolitan University, Summerstrand Campus, Department of Chemistry, University Way, Summerstrand, PO Box 77000, Port Elizabeth 6031, South Africa

## Abstract

In the title compound, C_7_H_9_NO, a derivative of benzyl alcohol, the endocyclic C—C—C angles are in the range 119.50 (12)–121.04 (12)°. In the crystal, mol­ecules are linked by N—H⋯O hydrogen-bond inter­actions, forming an extended two-dimensional framework parallel to *ab*. O—H⋯N inter­actions are also observed.

## Related literature

For the crystal structure of (3-(hy­droxy­meth­yl)phen­yl)-bis­(diphenyl­phosphinometh­yl)amine, see: Hursthouse *et al.* (2003 ▶). For the crystal structure of 3-nitro­benzyl alcohol as a co-crystal with platinum-containing coordination compounds, see: Oskui *et al.* (1999 ▶). For graph-set analysis of hydrogen bonds, see: Etter *et al.* (1990 ▶); Bernstein *et al.* (1995 ▶). For the use of chelating ligands in coordination chemistry, see: Gade (1998 ▶).
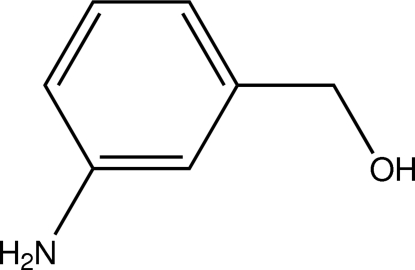


## Experimental

### 

#### Crystal data

C_7_H_9_NO
*M* *_r_* = 123.15
Orthorhombic, 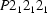

*a* = 4.7977 (4) Å
*b* = 6.2954 (6) Å
*c* = 21.6341 (18) Å
*V* = 653.42 (10) Å^3^
*Z* = 4
Mo *K*α radiation
μ = 0.09 mm^−1^
*T* = 200 K
0.53 × 0.47 × 0.19 mm

#### Data collection

Bruker APEXII CCD diffractometer
Absorption correction: multi-scan (*SADABS*; Bruker, 2008 ▶) *T*_min_ = 0.869, *T*_max_ = 1.000
6010 measured reflections
961 independent reflections
924 reflections with *I* > 2σ(*I*)
*R*_int_ = 0.020

#### Refinement

*R*[*F* ^2^ > 2σ(*F* ^2^)] = 0.033
*wR*(*F* ^2^) = 0.084
*S* = 1.11
961 reflections
94 parameters
H atoms treated by a mixture of independent and constrained refinement
Δρ_max_ = 0.19 e Å^−3^
Δρ_min_ = −0.16 e Å^−3^

### 

Data collection: *APEX2* (Bruker, 2010 ▶); cell refinement: *SAINT* (Bruker, 2010 ▶); data reduction: *SAINT*; program(s) used to solve structure: *SHELXS97* (Sheldrick, 2008 ▶); program(s) used to refine structure: *SHELXL97* (Sheldrick, 2008 ▶); molecular graphics: *ORTEP-3* (Farrugia, 1997 ▶) and *Mercury* (Macrae *et al.*, 2008 ▶); software used to prepare material for publication: *SHELXL97* and *PLATON* (Spek, 2009 ▶).

## Supplementary Material

Crystal structure: contains datablock(s) I, global. DOI: 10.1107/S1600536811029163/bx2363sup1.cif

Supplementary material file. DOI: 10.1107/S1600536811029163/bx2363Isup2.cdx

Structure factors: contains datablock(s) I. DOI: 10.1107/S1600536811029163/bx2363Isup3.hkl

Supplementary material file. DOI: 10.1107/S1600536811029163/bx2363Isup4.cml

Additional supplementary materials:  crystallographic information; 3D view; checkCIF report

## Figures and Tables

**Table 1:** Hydrogen-bond geometry (Å, °)

*D*—H⋯*A*	*D*—H	H⋯*A*	*D*⋯*A*	*D*—H⋯*A*
O1—H81⋯N1^i^	0.85 (3)	2.02 (3)	2.8620 (18)	171 (2)
N1—H71⋯O1^ii^	0.87 (2)	2.19 (2)	3.0588 (16)	177 (2)
N1—H72⋯O1^iii^	0.904 (19)	2.24 (2)	3.1204 (16)	165.3 (16)

Symmetry codes: (i) 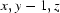; (ii) 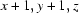; (iii) 
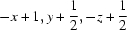
.

**Fig. 1.**
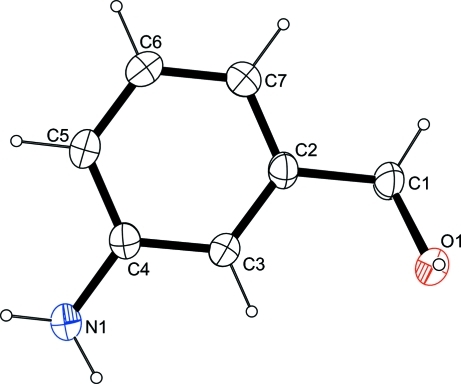
The molecular structure of the title compound, with atom labels and anisotropic displacement ellipsoids (drawn at 50% probability level).

**Fig. 2.**
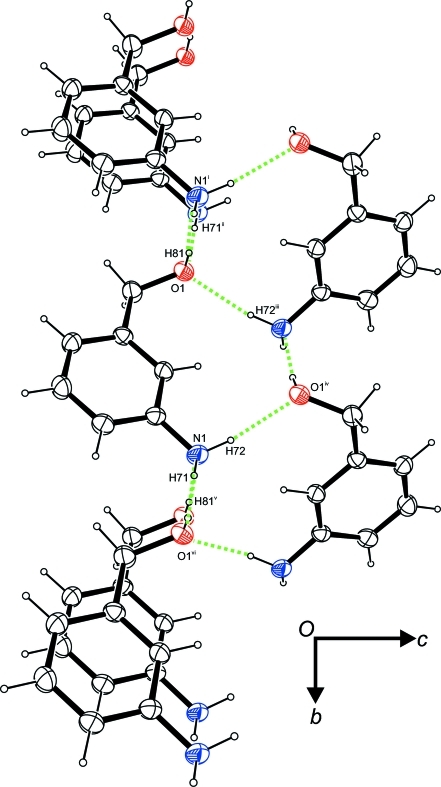
Intermolecular contacts, viewed along [-1 0 0]. Symmetry operators: ix, y - 1, z; iix - 1, y - 1, z; iii -x + 1, y - 1/2, -z + 1/2; iv -x + 1, y + 1/2, -z + 1/2; vx, y + 1, z; vix + 1, y + 1, z.

## supplementary crystallographic information

### Comment

Chelate ligands have found widespread use in coordination chemistry due to the enhanced thermodynamic stability of resultant coordination compounds in relation to coordination compounds exclusively applying comparable monodentate ligands (Gade, 1998). Combining two different donor atoms, a molecular set-up to accomodate a large variety of metal centers of variable Lewis acidity is at hand. In this aspect, 3-aminobenzyl alcohol seemed of interest due to its possible use as a strictly neutral or, depending on the pH value, as an anionic or cationic ligand. In addition, due to the set-up of its functional groups, it may act as mono- or bidentate ligand offering the possibility to create seven-membered chelate rings. To enable comparative studies in terms of bond lengths and angles in envisioned coordination compounds, we determined the molecular and crystal structure of the title compound. Information about the crystal structure of (3-(Hydroxymethyl)phenyl)-bis(diphenylphosphinomethyl)amine (Hursthouse *et al.*, 2003) as well as 3-nitrobenzyl alcohol as a co-crystallizate with platinum-containing coordination compounds (Oskui *et al.*, 1999) is available in the literature.

The hydroxymethyl group is not in plane with the aromatic system, the respective dihedral angle was found at 33.0 (2) °. Endocyclic C–C–C angles hardly deviate from the expected ideal values of 120 ° and range from 119.50 (12)–121.04 (12) °. The biggest angle is found on the C atom bearing the amino group (Fig. 1).

In the crystal structure, a cooperative set of hydrogen bonds involving all nitrogen- and oxygen-bonded hydrogen atoms is present. While the oxygen atom acts as twofold acceptor for hydrogen bonds exclusively stemming from the H atoms of the amino group, the nitrogen atom of the amino group serves as acceptor for a hydrogen bond originating from the hydroxyl group's O atom. In terms of graph-set analysis (Etter *et al.*, 1990; Bernstein *et al.*, 1995), the descriptor for this hydrogen bonding system on the unitary level is *C*^1^_1_(7)*C*^1^_1_(7)*C*^1^_1_(7). In the crystal the molecules are linked by N— H···O hydrogen-bond interactions, forming an extended two-dimensional framework parallel to the ab, Fig. 2. π-Stacking is not a prominent feature of the crystal structure with the shortest intercentroid distance between two aromatic systems measured at 5.5741 (10) Å .(Fig. 2).

### Experimental

The compound was obtained commercially (Aldrich). Crystals suitable for the X-ray diffraction study were obtained upon free evaporation of a solution of the compound in acetonitrile at room temperature.

### Refinement

Carbon-bound H atoms were placed in calculated positions (C—H 0.99-0.95 Å) and were included in the refinement in the riding model approximation, with *U*(H) set to 1.2*U*_eq_(C). The hydrogen atoms of the hydroxyl group as well as of the amino group were located on a difference Fourier map and refined freely.

### Crystal data

**Table:**

C_7_H_9_NO	*F*(000) = 264
*M**_r_* = 123.15	*D*_x_ = 1.252 Mg m^−^^3^
Orthorhombic, *P*2_1_2_1_2_1_	Mo *K*α radiation, λ = 0.71073 Å
Hall symbol: P 2ac 2ab	Cell parameters from 5183 reflections
*a* = 4.7977 (4) Å	θ = 3.4–28.1°
*b* = 6.2954 (6) Å	µ = 0.09 mm^−^^1^
*c* = 21.6341 (18) Å	*T* = 200 K
*V* = 653.42 (10) Å^3^	Platelet, brown
*Z* = 4	0.53 × 0.47 × 0.19 mm

### Data collection

**Table:**

Bruker APEXII CCD diffractometer	961 independent reflections
Radiation source: fine-focus sealed tube	924 reflections with *I* > 2σ(*I*)
graphite	*R*_int_ = 0.020
φ and ω scans	θ_max_ = 28.0°, θ_min_ = 3.4°
Absorption correction: multi-scan (*SADABS*; Bruker, 2008)	*h* = −6→6
*T*_min_ = 0.869, *T*_max_ = 1.000	*k* = −7→8
6010 measured reflections	*l* = −23→28

### Refinement

**Table:**

Refinement on *F*^2^	Primary atom site location: structure-invariant direct methods
Least-squares matrix: full	Secondary atom site location: difference Fourier map
*R*[*F*^2^ > 2σ(*F*^2^)] = 0.033	Hydrogen site location: inferred from neighbouring sites
*wR*(*F*^2^) = 0.084	H atoms treated by a mixture of independent and constrained refinement
*S* = 1.11	*w* = 1/[σ^2^(*F*_o_^2^) + (0.0484*P*)^2^ + 0.1019*P*] where *P* = (*F*_o_^2^ + 2*F*_c_^2^)/3
961 reflections	(Δ/σ)_max_ < 0.001
94 parameters	Δρ_max_ = 0.19 e Å^−^^3^
0 restraints	Δρ_min_ = −0.16 e Å^−^^3^

### Special details

**Table:**

Refinement. Due to the absence of a strong anomalous scatterer, the Flack parameter is meaningless. Thus, Friedel opposites (600 pairs) have been merged and the item was removed from the CIF.

### Fractional atomic coordinates and isotropic or equivalent isotropic displacement parameters (Å^2^)

**Table:**

	*x*	*y*	*z*	*U*_iso_*/*U*_eq_	
O1	0.3733 (2)	−0.37165 (17)	0.18291 (4)	0.0277 (3)	
H81	0.524 (6)	−0.439 (4)	0.1893 (9)	0.049 (6)*	
N1	0.8480 (3)	0.35877 (19)	0.19920 (6)	0.0287 (3)	
H71	1.002 (5)	0.432 (3)	0.1955 (9)	0.040 (5)*	
H72	0.811 (4)	0.303 (3)	0.2368 (9)	0.035 (5)*	
C1	0.3760 (3)	−0.2875 (2)	0.12190 (6)	0.0313 (3)	
H1A	0.1858	−0.2383	0.1114	0.038*	
H1B	0.4250	−0.4028	0.0927	0.038*	
C2	0.5769 (3)	−0.1056 (2)	0.11274 (6)	0.0236 (3)	
C3	0.6316 (3)	0.0359 (2)	0.16033 (6)	0.0252 (3)	
H3	0.5471	0.0147	0.1996	0.030*	
C4	0.8087 (3)	0.2089 (2)	0.15151 (6)	0.0237 (3)	
C5	0.9317 (3)	0.2381 (2)	0.09359 (7)	0.0303 (3)	
H5	1.0527	0.3552	0.0867	0.036*	
C6	0.8774 (3)	0.0967 (2)	0.04623 (7)	0.0325 (3)	
H6	0.9615	0.1177	0.0070	0.039*	
C7	0.7016 (3)	−0.0758 (2)	0.05529 (6)	0.0278 (3)	
H7	0.6667	−0.1727	0.0225	0.033*	

### Atomic displacement parameters (Å^2^)

**Table:**

	*U*^11^	*U*^22^	*U*^33^	*U*^12^	*U*^13^	*U*^23^
O1	0.0305 (5)	0.0253 (5)	0.0271 (5)	−0.0050 (5)	0.0030 (4)	0.0028 (4)
N1	0.0328 (6)	0.0248 (6)	0.0284 (6)	−0.0090 (5)	0.0021 (5)	−0.0024 (5)
C1	0.0372 (7)	0.0310 (7)	0.0259 (6)	−0.0156 (7)	−0.0035 (6)	0.0025 (5)
C2	0.0230 (6)	0.0219 (6)	0.0259 (6)	−0.0044 (5)	−0.0029 (5)	0.0024 (5)
C3	0.0274 (6)	0.0257 (6)	0.0225 (5)	−0.0066 (6)	0.0022 (5)	0.0013 (5)
C4	0.0239 (6)	0.0209 (6)	0.0265 (6)	−0.0022 (6)	−0.0011 (5)	0.0004 (5)
C5	0.0320 (7)	0.0275 (7)	0.0313 (7)	−0.0102 (6)	0.0051 (6)	0.0015 (6)
C6	0.0359 (7)	0.0359 (7)	0.0257 (6)	−0.0078 (7)	0.0071 (6)	0.0011 (6)
C7	0.0306 (6)	0.0281 (7)	0.0248 (6)	−0.0043 (6)	−0.0002 (6)	−0.0024 (5)

### Geometric parameters (Å, °)

**Table:**

O1—C1	1.4222 (16)	C2—C7	1.3922 (19)
O1—H81	0.85 (3)	C3—C4	1.3945 (19)
N1—C4	1.4105 (17)	C3—H3	0.9500
N1—H71	0.87 (2)	C4—C5	1.3972 (19)
N1—H72	0.904 (19)	C5—C6	1.382 (2)
C1—C2	1.5100 (19)	C5—H5	0.9500
C1—H1A	0.9900	C6—C7	1.389 (2)
C1—H1B	0.9900	C6—H6	0.9500
C2—C3	1.3865 (19)	C7—H7	0.9500
			
C1—O1—H81	109.2 (14)	C2—C3—H3	119.5
C4—N1—H71	113.4 (13)	C4—C3—H3	119.5
C4—N1—H72	111.9 (12)	C3—C4—C5	118.85 (12)
H71—N1—H72	117.0 (16)	C3—C4—N1	120.24 (12)
O1—C1—C2	114.20 (11)	C5—C4—N1	120.78 (12)
O1—C1—H1A	108.7	C6—C5—C4	120.04 (13)
C2—C1—H1A	108.7	C6—C5—H5	120.0
O1—C1—H1B	108.7	C4—C5—H5	120.0
C2—C1—H1B	108.7	C5—C6—C7	120.90 (13)
H1A—C1—H1B	107.6	C5—C6—H6	119.6
C3—C2—C7	119.66 (12)	C7—C6—H6	119.6
C3—C2—C1	120.72 (12)	C6—C7—C2	119.50 (12)
C7—C2—C1	119.58 (12)	C6—C7—H7	120.3
C2—C3—C4	121.04 (12)	C2—C7—H7	120.3
			
O1—C1—C2—C3	−33.0 (2)	C3—C4—C5—C6	−0.1 (2)
O1—C1—C2—C7	149.06 (13)	N1—C4—C5—C6	−176.05 (14)
C7—C2—C3—C4	0.4 (2)	C4—C5—C6—C7	−0.1 (2)
C1—C2—C3—C4	−177.54 (13)	C5—C6—C7—C2	0.5 (2)
C2—C3—C4—C5	0.0 (2)	C3—C2—C7—C6	−0.6 (2)
C2—C3—C4—N1	175.95 (13)	C1—C2—C7—C6	177.36 (13)

### Hydrogen-bond geometry (Å, °)

**Table:**

*D*—H···*A*	*D*—H	H···*A*	*D*···*A*	*D*—H···*A*
O1—H81···N1^i^	0.85 (3)	2.02 (3)	2.8620 (18)	171 (2)
N1—H71···O1^ii^	0.87 (2)	2.19 (2)	3.0588 (16)	177 (2)
N1—H72···O1^iii^	0.904 (19)	2.24 (2)	3.1204 (16)	165.3 (16)

Symmetry codes: (i) *x*, *y*−1, *z*; (ii) *x*+1, *y*+1, *z*; (iii) −*x*+1, *y*+1/2, −*z*+1/2.
